# Splash-free urinals for global sustainability and accessibility: Design through physics and differential equations

**DOI:** 10.1093/pnasnexus/pgaf087

**Published:** 2025-04-08

**Authors:** Kaveeshan Thurairajah, Xianyu (Mabel) Song, J D Zhu, Mia Shi, Ethan A Barlow, Randy C Hurd, Zhao Pan

**Affiliations:** Department of Mechanical and Mechatronics Engineering, University of Waterloo, 200 University Ave W, Waterloo, ON N2L 3G1, Canada; Department of Mechanical and Mechatronics Engineering, University of Waterloo, 200 University Ave W, Waterloo, ON N2L 3G1, Canada; Department of Mechanical and Mechatronics Engineering, University of Waterloo, 200 University Ave W, Waterloo, ON N2L 3G1, Canada; Department of Mechanical and Mechatronics Engineering, University of Waterloo, 200 University Ave W, Waterloo, ON N2L 3G1, Canada; Department of Mechanical Engineering, Weber State University, 1465 Edvalson St, Ogden, Utah 84403, USA; Department of Mechanical Engineering, Weber State University, 1465 Edvalson St, Ogden, Utah 84403, USA; Department of Mechanical and Mechatronics Engineering, University of Waterloo, 200 University Ave W, Waterloo, ON N2L 3G1, Canada

**Keywords:** urinal, splash, droplet, sustainability

## Abstract

Urinals are a staple of public spaces yet their designs have remained essentially stagnant for over a century. The use of urinals often results in significant splatter (splashback) as urine splashes upon impact with the urinal generating droplets which travel back onto the floor and user, which generates unhygienic environments, high cleaning costs, and adds unpleasant workload for custodial staff. Impinging stream angle is one of many factors that affect splashback. We theoretically predict and experimentally validate that when the impinging angle is below an invariant critical value of $∼30^{\circ }$, the flow rate of splashback under human urination conditions can be significantly suppressed. We propose novel urinal designs that were generated by solving differential equations derived from the isogonal curve problem to ensure the urine stream impacts at or below this critical angle. Experiments validate that these designs can substantially reduce splashback to only 1.4% of the splash of a common contemporary commercial urinal. The widespread adoption of the urinal designs described in this work would result in considerable conservation of human resources, cost, cleaning chemicals, and water usage, rendering large-scale impacts on modern society by improving sustainability, hygiene, and accessibility.

**Significance Statement**

Hygiene infrastructure has transformed human life but is often overlooked due to its ubiquity. The male urinal, for example, its design has remained stagnant for over a century. Existing designs generate significant splashback (on the order of $10^{6}$ L/day in the United States alone), creating hygiene issues of their own, and requiring human and material resources to clean. We apply fluid physics to develop and experimentally confirm a theoretical model of splash suppression on contoured surfaces. This model is then applied to create splashless urinals. Tests show that our designs eliminate splashback, thereby reducing the resources consumed in cleaning while improving hygiene in public spaces. Global adoption of our designs renders large-scale improvements to sustainability, sanitation, and accessibility.

## Introduction

Public restrooms are an important, though commonly disregarded, part of human history. The incorporation of adequate sewer systems eliminated plagues, and transformed city life (1). The urinal is a near-universal feature of the modern public bathroom—space efficient and fast—urinals are an ideal solution for at least half the population when they void. Urinals made an early appearance historically with examples elaborately carved from stone; instances of these receptacles have been found in Monastaries in the ancient capital of Sri Lanka built around 1,000 CE (2, 3). Modern urinals emerged in the 19th century to serve industrial workers in Europe (4). Paris alone had over 1,000 “Pissoirs” in the 1900s (4). The urinal has changed little in over a century. The urinal in Marcel Duchamp’s paradigm-altering art piece “La Fontaine” (1917) would not seem out of place in a modern restroom. This stagnation in design does not mean that the modern urinal has been perfected. Splashback—droplets of urine on the floor or user originating from splashing on the urinal surface, remains a significant problem associated with urinals and contributes to unsanitary and unpleasant conditions in public restrooms.

**Fig. 1.**
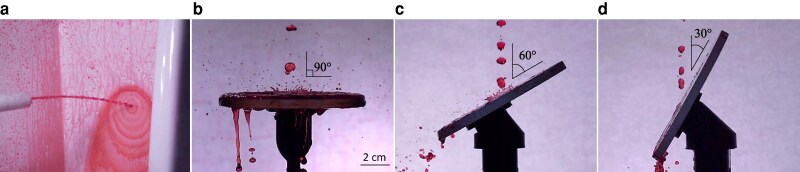
a) High-speed image of splash in a common contemporary commercial urinal. b–d) High-speed images of splash events generated by flow from an anatomically accurate nozzle impacting a glass disk at an impinging angle of $90^{\circ },60^{\circ }$, and $30^{\circ }$, respectively.

Although mostly sterile, urine provides a fertile breeding ground for bacteria and contributes to foul odors common in public restrooms (5, 6). Aerosolized droplets can spread far, coating large areas of the floor and often the users themselves (see Figs. 1a and 5b for reconstructions of a typical splashback event when using a common commercial urinal). For example, with around 56 million urinals in nonresidential settings in the United States alone, more than $0.35\times 10^{6}$ L, on the order of 1 million L, of urine is splashed onto the floor daily (see Supplementary material and Ref. (7)). Urine splashback also poses a notable health risk to washroom occupants. The surfaces of urinals have significantly higher concentrations of bacteria than traditional toilets, with surrounding floors having the highest level (8). Ammonia levels on the floor surrounding the urinal are on the order of 100 times higher than any area near a traditional toilet—indicating significant splashback, and providing an ideal environment for the aforementioned bacterial colonies (9).

The hygiene issues caused by splashback contribute to high cleaning costs. For example, the Toronto subway was projected to spend an annual average of $122,418.18 in Canadian dollars (CAD) per bathroom on cleaning costs alone from 2020 through 2024 (10). Using the cost per bathroom associated with the Toronto subway as an estimate, up to around $10,000 CAD per bathroom could be saved annually. Although savings vary regionally, the large number of bathrooms and urinals globally ensures enormous total savings. Furthermore, such cleaning is intensive in water and cleaning solvent usage, as well as cleaning tool replacement. Given the large number of bathrooms across the world (11), this nearly invisible splashback produces high financial and environmental costs.

Such consumption of resources adds to the larger issue faced worldwide of water stress, especially by urban regions (12, 13). Urinal splashback also creates a constant need for cleaning which requires janitorial personnel to regularly clean up foul-smelling human waste and repeatedly disinfect affected surfaces. The elimination of splatter from restrooms has the potential to reduce the amount of human labor necessary for public restroom maintenance in a manner comparable to the modern washing machine drastically reducing the drudgery once associated with washday (14).

Limited attempts have been made to fight splashback. Urinal screens and mats have been developed to attempt to mitigate this problem (15, 16). Some such models are placed within the urinal and others on the floor—simply catching rather than preventing splashback. Such efforts are hard to quantify with no reporting on their effectiveness in splash prevention, and no ideal solution yet exists. At Amsterdam’s Schiphol airport, the use of fly stickers to change aiming behavior resulted in a reportedly 50–80% reduction (not elimination) in splashback observed and an 8% reduction in cleaning costs (17).

To address the consequences of urine splashback on hygiene, water consumption, and human labor, we designed alternative urinals that eliminate splashback. We proceed to validate the new designs using both computational and experimental methods. The results demonstrate that our urinal designs reduce the formation and spread of satellite droplets from an incoming stream compared to existing urinals. These findings offer encouraging evidence that nondrastic, but precisely devised design changes to common fixtures, such as the urinal, can result in scalable improvements to user health and global sustainability.

## Results

### Nonsplash critical impinging angle

Splash generated from a jet or droplet train impinging on a flat surface is a complex phenomenon depending on many factors, including the impact speed (*U*), impinging angle (*θ*), dynamic viscosity (*μ*), density (*ρ*), diameter of the jet or droplet (*D*), surface tension (*γ*) of the liquid, ambient pressure (*P*), as well as the wettability, contact angle, and roughness of the surface. The impinging angle is measured between the incoming jet or droplet train and a line tangent to the surface as seen in Fig. 1. Nondimensional numbers quantify the relative importance of these factors; for example the Reynolds (Re=ρUDμ), capillary ($\mathrm{Ca}=\frac{\mu U}{\gamma }$), and Weber ($\mathrm{We}=\frac{\rho U^{2}D}{\gamma }$) numbers (18, 19). In the context of urination, most of these factors cannot be changed. However, the impinging angle can be controlled to reduce or eliminate splash.

Recent studies show that splash does not occur when the impinging angle falls below a certain critical threshold depending on the We (18). We extended this work to develop a theoretical model predicting the reduction in splash flow rate as the impinging angle drops. We defined the splash ratio, $Q^{*}$, as splashed mass divided by total impinged water mass. $Q_{90^{\circ }}^{*}$ is the nominal value of $Q^{*}$ for a given flow condition impinging perpendicular to the surface, used to normalize the splash ratio. To better predict $Q^{*}/Q_{90^{\circ }}^{*}$, we introduce a modified Weber number ${\mathrm{We}}^{†}=\mathrm{We}\sin^{2}\;\theta$, which is dictated by the velocity normal to the surface that the droplet impacts (where *θ* is the impinging angle of the droplet). Our results indicate that the relative splashed volume has an almost invariant inflection point independent of We and Re under conditions of human urination (see Methods section). The model predicts that at $\theta =30^{\circ }$, the splash is reduced by about 95% from maximum splash. This implies the possibility of designing a urinal geometry so that the impinging angle is always at or below this critical angle; consequently eliminating splash regardless of factors dependent on the user.

**Fig. 2.**
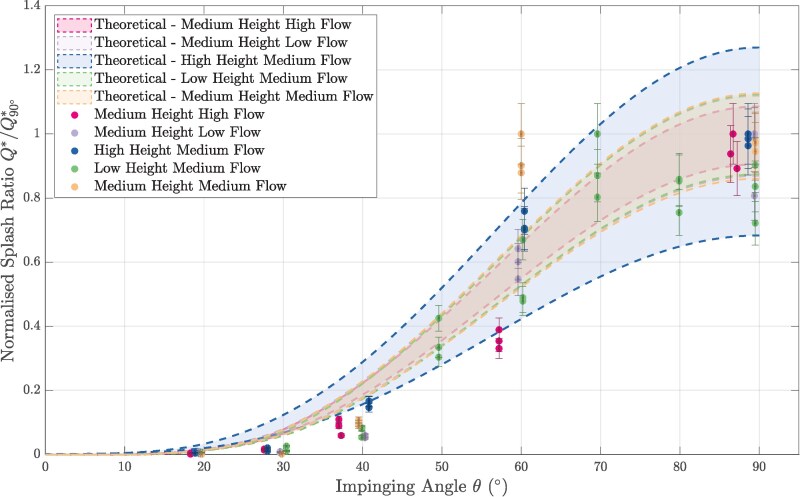
Normalized splash ratio vs. impinging angle for theoretical predictions and experimental measurements. Different flow conditions created by varying the height between the nozzle and the target plate as well as flow rate. Flow conditions tested are: (i) medium height high flow, (ii) medium height low flow, iii) high height medium flow, (iv) low height medium flow, and (v) medium height medium flow. Error bars represent uncertainty associated with experimental instrumentation. Shaded patches represent the prediction based on the theoretical model for a range of droplet diameters 1 SD above and 1 SD below the mean observed in the experiments. The center lines for each patch are not shown to improve visualization.

To verify the theoretical model and find the critical angle $\hat{\;\theta }$ relevant to human urination, we measured the splash generated by a jet of water from an anatomically accurate nozzle above an inclined glass plate and varied the angle of inclination of the plate *θ* from $20^{\circ }$ to $90^{\circ }$ measured from the impinging stream. As seen in Fig. 2, the experimentally measured normalized splash ratio ($Q^{*}/Q_{90^{\circ }}^{*}$) produces good agreement with the theoretical model. This is also visualized in images of the tests in Fig. 1b–d where the splash formed upon impact decreases with the impinging angle, and is nearly zero at the critical value. It is also interesting to observe that when a dog urinates on a tree or vertical wall, the impinging angle of the stream is generally shallow, which would reduce splashback back onto the dog’s fur. Although dogs urinate on trees to mark their territory, low splash is an unintended but positive outcome (20). The observation of a critical angle provides a design criterion to meet in the development of a splashless urinal.

### Urinal basin profile designed by solving differential equations

Our approach to design a nonsplash urinal requires that the 3D stream impinges the urinal surface at or under the critical angle (i.e. $\theta \leq \hat{\;\theta }$). This complex 3D problem can be simplified by instead requiring the 2D projection of the urine stream to impinge at the critical angle:


(1) $$\theta =\hat{\;\theta }=30^{\circ }.$$

**Fig. 3.**
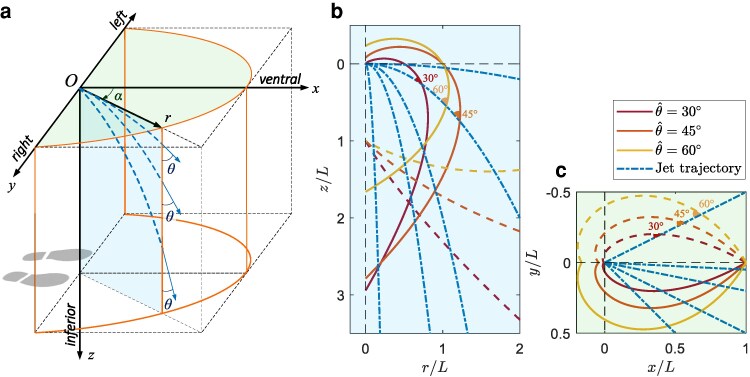
a) Coordinate system used to define the urinal problem. The top view plane is shaded in green and the side view is blue. b) The blue dash-dotted lines show different parabolas representing the trajectories for urine streams. The generated isogonal curves (solid or dashed) in the vertical plane are shown in scarlet, orange, and yellow for $30^{\circ }$, $45^{\circ }$, and $69^{\circ }$, respectively. c) The top view isogonal curves for various angles shown in scarlet, orange, and yellow. Blue dash-dotted lines show the projection of urine trajectories onto the plane as rays intersecting the curves at constant angles. For each angle, two curves are shown (one solid and one dotted) which intersect at $\alpha =0^{\circ }$ forming a mathematical cusp. The solution is nondimensional with *L* representing a characteristic length scale. The parabolae are self similar as are the isogonal curves shown which can be scaled as required.

The 3D problem is described using a cylindrical coordinate system (*r*, *α*, *z*, seen in Fig. 3a). Two different 2D projections can be defined: a side view (i.e. *r–z* plane in Fig. 3a and b) where the stream is a family of parabolic curves varying over the course of urination as bladder pressure drops, and a top view (i.e. *x–y* plane in Fig. 3a and c) where the stream forms rays depending on user aim. Satisfying (1) leads to solving for isogonal curves in each plane to intersect the urine trajectory at a constant angle.

**Fig. 4.**
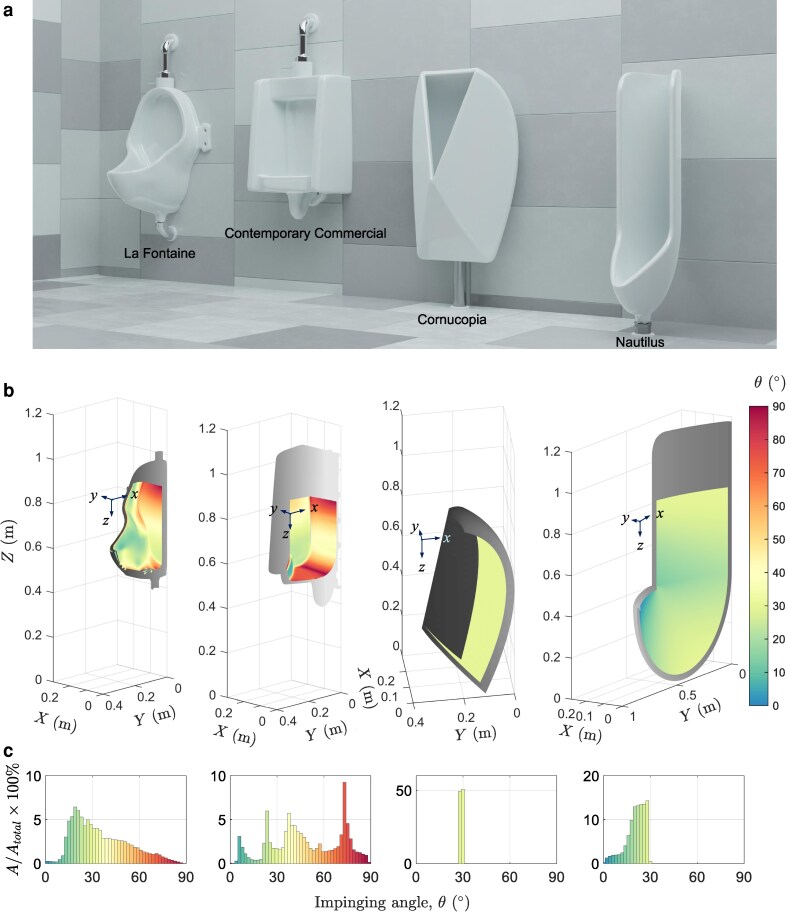
a) 3D renderings of urinals. From left to right: Duchamp’s “La Fontaine,” contemporary commercial model, Cornucopia, Nautilus. b) Maps of impinging angles on urinals and c) histograms showing percent of the area at various impinging angles (*A* is the area where the impinging angle falls into a certain range and $A_{\mathrm{total}}$ is the total area that can be impinged on the inner urinal surface).

The side view projection results in a curve given by:


(2) $$C=\frac{3}{\sqrt{8\Theta^{2}-1}}\text{arctan}\left(\frac{4\Theta z-r}{r\sqrt{8\Theta^{2}-1}}\right)+\frac{1}{2}\ln\;\left(2\Theta z^{2}-rz+\Theta r^{2}\right),$$

where $\Theta =\pm \tan\;\hat{\;\theta }$, and *C* is a constant that is determined by an auxiliary or boundary condition associated with the installation of the urinal. Variations of this curve for different values of $\hat{\;\theta }$ are shown in Fig. 3b. The curves obtained using $\hat{\;\theta }=30^{\circ }$ resemble the cross-section of a cornucopia. Revolving these curves about the *z*-axis generates a 3D internal surface of one urinal design which the research team named the “Cornucopia” (see Fig. 4a, third from left).^a^

The isogonal curve problem in the top view projection produces logarithmic spirals—the same curve as present in the shell of a nautilus:


(3) $$r=B\exp\;\left(\frac{\alpha }{\Theta }\right),$$

where *B* is a constant that controls the scale of the curve. Different logarithmic spirals for different $\hat{\;\theta }$ are seen in Fig. 3c. By extruding this curve, a 3D urinal surface profile can be designed and dubbed the “Nautilus” as seen in Fig. 4a (far-right urinal).

The isogonal nature of these designed surfaces is visualized in Fig. 4b. Impinging angles were mapped onto urinal surfaces for both existing and newly designed models assuming a user with median male inseam height and typical installation (21). Both Duchamp’s “La Fontaine” and a contemporary commercial design are not universally poor, but have areas where impinging streams have high angles, and thus high splashback. The novel designs, Cornucopia and Nautilus, have impinging angles at or below $30^{\circ }$ across their entire area. The relative distribution of impinging angles on the urinal surfaces is seen in Fig. 4c.

### Experimental validation

#### Qualitative tests: splash pattern

**Fig. 5.**
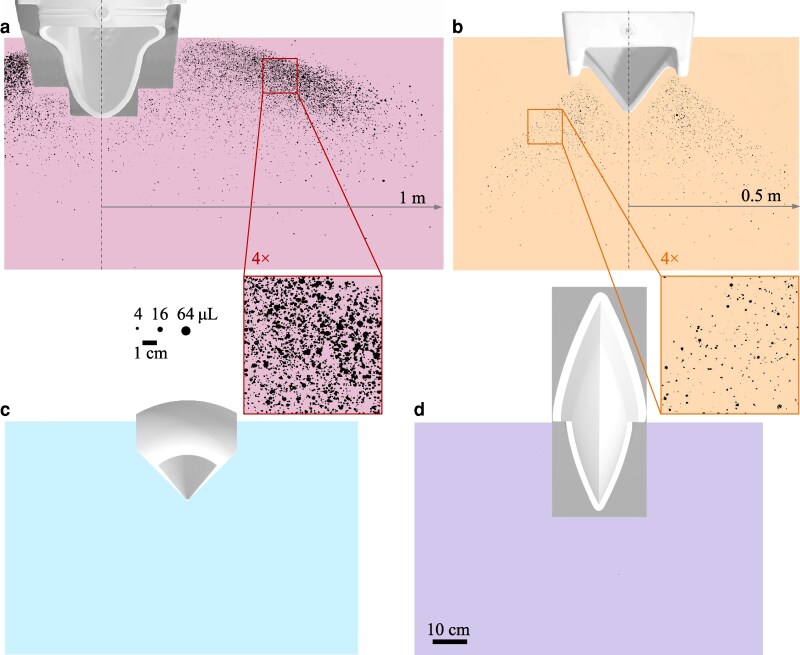
Images of splatter generated by each urinal under the medium user height, high flow rate test condition with a total “urinated” volume of 1 L: a) La Fountaine, b) contemporary commercial, c) Cornucopia, and d) Nautilus. The gray visualizes the top plane projection of the foam urinal model used in the splatter tests, whereas the white shows the same projection of the ceramic urinal as it would be installed. The stains from sessile droplets of known volumes are indicated at the same scale as the zoomed sections.

The performance of the four urinals was evaluated qualitatively, first, through observation of the splatter on the floor around the urinals. A pseudo-urethra nozzle matching the internal geometry of a human urethra (22) was used to “urinate” a controlled jet of dyed water onto urinals and the subsequent splash was caught on a large paper on the floor. More details about the urination apparatus can be found in Section S2. All urinals were tested with identical volumes of water, flow rates (representing a typical high flow rate), and median user height (23, 24). The resulting images were binarised and shown in Fig. 5. Both novel designs perform well, with nearly no observable splash compared to the contemporary commercial urinal as well as Duchamp’s “La Fontaine.” Existing designs generate splatter out to extensive range, with droplets reaching as far as 1 m to either side of “La Fontaine” and ∼0.5 m for a contemporary commercial urinal.^b^

#### Quantitative tests: splash ratio

**Fig. 6.**
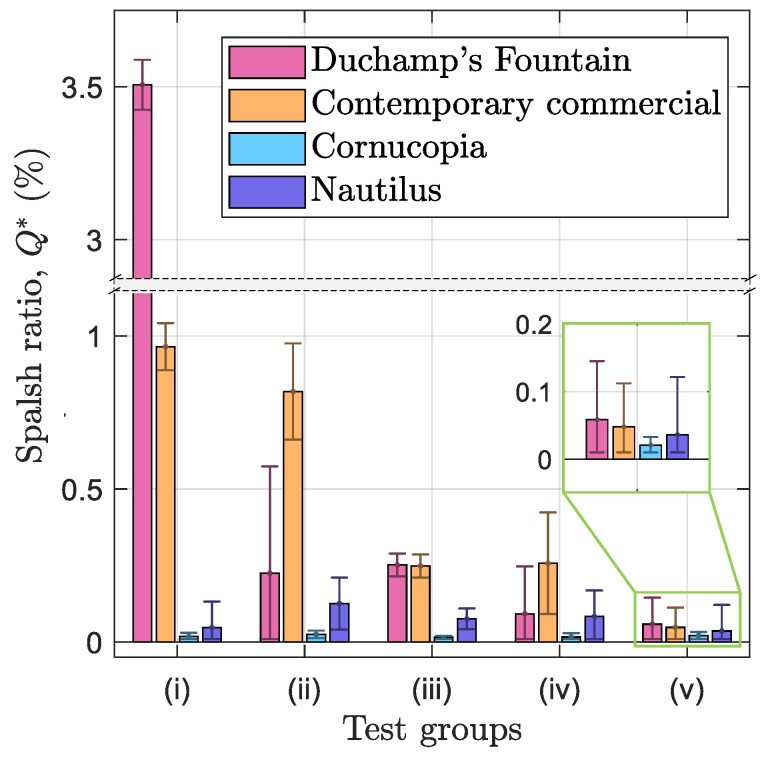
Splash ratio on all urinals under various test conditions. All urinals are at standard installation height. Test conditions are the same as in Fig. 2, namely: i) medium height high flow, ii) medium height low flow, iii) high height medium flow, iv) low height medium flow, and v) medium height medium flow. All error bars represent 2 SD from three independent measurements combined with uncertainty contributed from experimental error.

Building on qualitative results, quantitative data were obtained by measuring the mass fraction of splashed water resulting from “urination” from the aforementioned urination apparatus. Splash ratio $Q^{*}$ was used to assess the performance of different urinals. Significant reductions in splash are observed as seen in Fig. 6. The novel urinal designs consistently outperform both the contemporary commercial urinal as well as the older “La Fontaine” model. While $Q^{*}$ is reduced, the reduction achieved varies between test conditions. Whereas existing designs see jumps in splash under certain conditions, the novel designs have consistently low $Q^{*}$.

Notably the least improvement is seen under the medium flow medium height test condition, with the Nautilus design reducing $Q^{*}$ by 24% compared to the contemporary commercial urinal. However, although the reduction is less spectacular compared to other test conditions, this condition produced low splash overall. The contemporary commercial design performs poorly with low or high flow rate conditions reflecting the high angle regions observed in Fig. 4b. Under these normally high splash conditions the same Nautilus urinal achieves 85% and 95% reductions in splash, respectively; reducing splash where it occurs most. The performance of the Cornucopia urinal, in suppressing splash, is even higher.

The results for the century-old design of Duchamp’s “La Fontaine” exemplify the limited improvements seen in the splashback reduction to date. The flat bottom should generate a high impact angle at low stream velocities as seen in Fig. 4b, yet the surrounding walls catch most of these droplets. This is not the case for the contemporary commercial design, and this modern urinal underperforms under such conditions. In the high flow condition, however, “La Fontaine” generates more splash than any other urinal and flow condition. This is largely due to the shallow walls on the sides of the urinal. Although the high impact angle is also present in the contemporary commercial urinal, the deeper walls catch a larger fraction of these droplets. This blocking effect proves significant in offsetting splash where the impact angle would otherwise suggest more splashback. This effect also helps explain the strong showing of the Cornucopia design. The extensive side walls and deep rim catch the few droplets that are generated and further improve performance. The measurable splash mass observed in the quantitative tests contrasts with the nearly blank splash patterns in the qualitative data. A possible explanation for this discrepancy is that only droplets of small diameter are generated which evaporate prior to contact with the ground. Finally, we want to emphasize that the prototypes used for testing are made from foams coated by polished resin, which has a contact angle of about $60^{\circ }$. This low wettability is unfavorable in terms of reducing splash (18, 19). If the urinals for testing were made from porcelain with a low contact angle (or adopting a more conservative critical angle (e.g. $\hat{\;\theta }=20^{\circ }$) while using hydrophobic surfaces), even greater reductions in $Q^{*}$ can be obtained (see Supplementary materials).

### Analysis of designs

Both novel urinal designs achieve significant splash reduction, with splash mass data, splash patterns, and impinging angle renders, all showing consistently low splash across multiple urination conditions. The Nautilus design comes out as the ideal choice due to the following factors. First, common urinals are subject to an installation dilemma. If installed at a typical height, it is not accessible to those with a short stature including children; while if installed at a lower height to address accessibility, it becomes uncomfortable for most people (adults) and generates greater splash due to larger drop distance and thus higher impact speed. The Nautilus’s low lip of between 430 mm and 140 mm (possibly even lower depending on stylistic choices) is accessible to users of all heights (even in a wheelchair), with the design exceeding accessibility standards which require a lip no more than 430 mm off the floor. (25, 26). Furthermore, the larger open area allows for significantly easier cleaning, a practical design aspect, which cannot be overlooked.

The Nautilus tolerates poor aim and as such it may prove beneficial on aircraft and boats where the stability cannot be taken for granted. The Cornucopia is a mathematically ideal design given a user with a certain height but, as with many existing urinals, is sensitive to user height, and therefore, it is not suitable for all users.

## Discussions

By leveraging the impact dynamics of water droplets, we have created a novel urinal design that nearly eliminates splashback generated by urinal use, far exceeding the performance of contemporary urinals. The substantial suppression of urine splashback reduces the utilization of water, cleaning chemicals, and human resources required for the maintenance of public washrooms. If the novel Nautilus design replaced the over 56 million urinals in nonresidential settings in the United States alone, we conservatively estimate that on the order of $10^{6}$ L of urine would be prevented from being splashed onto the floor daily. Assuming 10 times as much water is required to clean a particular volume of splashed urine, we can save up to 10 million L of fresh water used for cleaning per day.

The minimization of maintenance requirements reduces the financial and environmental costs of operating public washrooms. In addition to cost savings, operators benefit from greater client, employee, and customer satisfaction from a clean and healthy environment. Those who do not use urinals, and as gender neutral washrooms proliferate, also benefit from cleaner bathroom space. The accessibility of washrooms will be improved with the implementation of our urinals which exceed all established accessibility standards in every installation. The novel urinal proposed in the present research reflects refreshing functional designs for a staple device, which has stagnated for a century. The superior performance of our design is achieved solely by manipulating the geometry of the urinal and does not require incorporating expensive or complicated surface materials or coatings; thus the design is compatible with conventional materials and immediately suitable for mass implementation. Further performance may be obtained, for example by using a soft surface to suppress splash, or hydrophobic surfaces for a both splashless and waterless design (27). Considering the large number of urinals worldwide (at the scale of one billion (11)), global adoption of the proposed designs delivers large-scale benefits to a more sustainable and accessible society.

The model describing changing splash with impinging angle has potential for further utility beyond the urinal case studied here. Many processes exist where minimizing splash is a desirable outcome. Future work is required to establish the validity of this model with various fluids with different properties (i.e. viscosity, surface tension, density), across a wide range of We and Re.

### The “urine-no,” a hostile antiurination surface

**Fig. 7.**
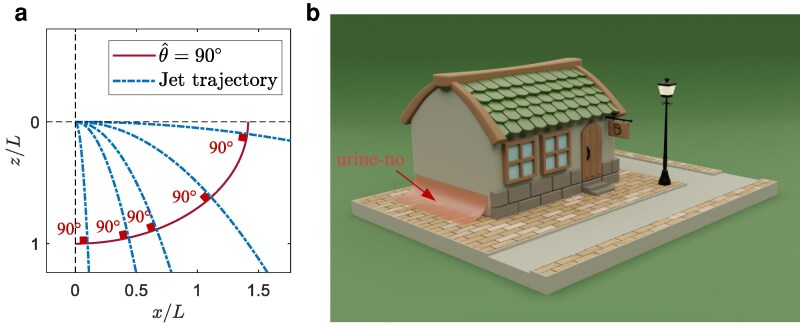
a) The generated isogonal curve (solid scarlet line) in the vertical plane for an impinging angle of $90^{\circ }$. *L* is the inseam height of users, as an characteristic length, to normalize the scale of the curve. b) A rendering of the “urine-no” installed on the exterior of a building to deter public urination. The surface is generated by extruding the curve in (a).

Instead of minimising splash, it can instead be maximized by setting $\hat{\;\theta }=90^{\circ }$, see Fig. 7a. Although not suitable as a commonly practical urinal, it showcases the design philosophy. Such a surface could be installed outdoors to deter public urination as the offender would fall prey to enhanced splash-back. This hostile surface may be dubbed as “urine-no,” as shown in Fig. 7b.

## Methods

### Generating urinal profiles

To generate the described urinal surfaces, urine trajectory can be projected onto either the top-view x–y plane or the side-view *r–z* plane (see Fig. 3a). Satisfying (1) leads to solving for isogonal curves to intersect the urine trajectory at a constant angle.

For the projection onto the *r–z* plane, assuming the initial direction of a urine jet is horizontal, the projectile motion of the fluid can be described by a family of parabolae: $z=g(r)=kr^{2}$, where k∈(0,∞) is a parameter that determines the shape of the trajectory (blue dashed curves in Fig. 3b). The limit of k→0 corresponds to the initial stage of urination, where bladder pressure and, consequently, jet velocity are high and the stream is almost horizontal. While k→∞ indicates the trajectory of a droplet train at the end of the urination, when the droplets fall almost vertically. The goal is to design curve(s) z=f(r) that intersects z=g(r) by $\hat{\;\theta }$ for arbitrary *k*, which can be modeled by the ordinary differential equation:


(4) $$\Theta =\frac{2f(r)-rf^{\prime }(r)}{r+2f(r)f^{\prime }(r)},$$

where $\Theta =\pm \tan\;\;\hat{\;\theta }.$ The solution of (4) is an implicit function given in (2).

The problem can also be solved in the *x–y* plane projection. In this case, the stream forms a family of rays starting from the origin *O*, pointing in arbitrary angles *α*. To design an isogonal curve intersecting the rays at a constant angle, the following differential equation is established in polar coordinates:


(5) $$\frac{dr}{d\alpha }=\frac{r}{\Theta },$$

and the solution is the classic logarithmic spiral as seen in (3).

### Impinging angle analysis

In order to generate impinging angle maps, urinals were modeled as a 3D surface composed of triangular facets. The urine streams were simulated by parabolae initiated at the urethral outlet and ending at the center of each facet. The parabolae start horizontally and the angle between the tangent of the parabola and the normal vector of the surface was measured. This was repeated for all facets making up the inside surface of the urinal to generate an angle map.

### Critical angle testing

The critical angle was measured experimentally by quantifying the mass fraction of water that splashed upon impact with a glass disk. A urination-simulating apparatus was set up using a 3D printed nozzle with an anatomically accurate urethra geometry, pump, flow rate meter, flow totalizer, and valves. The nozzle was oriented vertically downward above the glass disk. The angle of the disk, relative to the impinging stream, was adjusted to vary the impinging angle *θ*.

The nozzle height above the disk was changed according to the test conditions in the context of human urination. Three flow rates of 0.4, 0.7, and 1.9 L/min (low, medium, and high flow, respectively, in the text) were tested along with three nozzle heights of 184, 154, and 933 mm (low, medium, and high heights, respectively); allowing for a range of conditions from the lowest flow and slowest jet to the most extreme conditions expected with urination. Given sufficient time the jet will break up into droplets due to Rayleigh-Plateau instability. Median droplet diameters were measured using a series of photographs and ranged from 4.4 mm to 7.2 mm. The Weber numbers associated with these median droplet diameters ranged from 340 to 1,400. The specific test matrix and justifications can be found in the Supplementary material. The disk was 10 cm in diameter and itself placed within a bucket with an inner diameter of 18.5 cm. Splash outside of the bucket was caught with a paper towel, with the mass measured before and after each test. The relative humidity was maintained above 60% to prevent the droplets from drying within the course of the test. A flow totalizer automatically counted the volume throughput and was used to determine the volume of water used in each test.

### Theoretical model development

Upon impact with a surface, a droplet forms lamella which moves radially outwards; the lamella experiences aerodynamic lift and subsequently splash (18). We extended existing work by assuming that as more lift is required for more ejecta, the volume of splash scales to surplus lift over the threshold. The splash fraction scales with average suprlus lift and was expressed in the following integral:


(6) $$Q^{*}∼\frac{1}{\pi }\int_{0}^{\phi^{*}}V_{l}^{2}-{V_{l}^{*}}^{2}\;d\phi ,$$

where $V_{l}$ is the lamellar velocity, $V_{l}^{*}$ is a threshold lamellar velocity, and $\phi^{*}$ is a critical angle associated with $V_{l}^{*}$, beyond which, no splash occurs.

The integral evaluates to the following expression with the derivation available in the Supplementary material:


(7) $$\begin{array}{rl} \frac{Q^{*}}{Q_{90^{\circ }}^{*}} & =\frac{1}{\pi }\frac{\sin\;\theta -{v^{*}}^{10/3}}{1-{v^{*}}^{10/3}}\\ & \;+\frac{\phi^{*}\left(\cos^{2}\;\theta \left(2+\sin\;2\phi^{*}-3{\beta^{†}}^{4/3}\right)+\frac{2\sqrt{3}}{3\phi^{*}}\sin\;2\theta {\beta^{†}}^{2/3}\sin\;\phi^{*}\right)}{3\pi \beta^{4/3}\left(1-{v^{*}}^{10/3}\right)}, \end{array}$$

where


$$\phi^{*}=ℜ\left[\cos^{-1}\;\left(\sqrt{3}\beta^{2/3}\frac{{v^{*}}^{5/3}-\sin^{5/3}\;\theta }{2\cos\;\theta }\right)\right],$$

$\beta =1.1\sqrt{We}$ , $\beta^{†}=\beta \sin\;\theta ,$  $\beta^{*}=v^{*}\beta$, and $v^{*}=V^{*}/V_{0}$ is a nondimensional critical velocity. $V_{0}$ is the incoming droplet velocity. $V^{*}$ and ${\mathrm{We}}^{*}$ represent critical values for a normal impact. We chose $V^{*}=0.89$, corresponding to the criterion $\sqrt{\mathrm{Ca}}=0.35$ introduced by Vander Wal et al. (28), Eq. (7) gives the prediction of the normalized splash ratio.

### Urinal production

Urinals for testing were prepared by shaping high-density foam using a computer numerical controlled mill. Our machining setup was highly accurate for the inside surface of the urinal, however, is limited in manufacturing the outside surfaces effectively. The foam was coated in epoxy resin before being sanded, painted, and waxed to provide a smooth waterproof surface. This method was used to prepare all urinals used in testing with the exception of the contemporary commercial urinal which was purchased ready-made out of glazed ceramic.

### Splashback quantification

The urinals were evaluated with the same urination apparatus consisting of a 3D printed anatomically accurate nozzle, same for all tests, as used in the critical angle testing. The urinals were installed according to ASME standards and the nozzle was placed horizontally at various inseam heights (21). The only exception was the high height condition for the Cornucopia urinal, where the nozzle was tilted downwards at an angle of $15^{\circ }$ to ensure that the urine stream fell within the urinal rather than overtop. Splashed droplets were measured by paper towel hung using clips from a steel wire frame enclosing the urinal. The paper towel was weighed and the relative humidity was managed the same way as the critical angle testing. All tests were conducted with a nominal water volume of 1 L. Five conditions were tested, and the parameters can be found in the Supplementary material. Three were at median inseam height for an American male (86.6 cm) with standard installation of urinals and the same flow rates as in Critical angle testing section 4.3. Two were conducted with 93.3 and 79.6 cm, corresponding to 95th and 5th percentile inseam height, respectively, both at medium flow (0.7 L/min) (23).

### Splatter visualization

The splatter visualizations were generated with the same urination apparatus, but with colored water. The nozzle was placed at the medium height and set to the high flow rate. The nominal volume of water “urinated” was 1.0 L. The splashback was caught on a large piece of lightly wicking “kraft” paper and left to dry before the paper was imaged. The images were postprocessed by thresholding RGB values of the splatter patterns and separating them from the background with pseudo-color. Sessile droplets of known volume were placed on identical paper and imaged to provide a reference scale.

## Supplementary Material

pgaf087_Supplementary_Data

## Data Availability

The data underlying this article are contained in the article and Supplementary material.

## Notes

a This design process can also be used to design a “hostile” surface to deter urination in public areas (e.g. walls outside a pub, dubbed “ urine-no”) by maximizing impinging angle and thus splash back (see Supplementary material).

b This experiment can be thought of as a reconstruction of a bathroom break at work a century ago in Paris, which perhaps gives additional meaning to the title “Fountain.”
