# Supplementary material for: Splash-free urinals for global sustainability and accessibility: Design through physics and differential equations
Source: PNAS Nexus. 2025 Apr 8;4(4):pgaf087. doi: 10.1093/pnasnexus/pgaf087 (PMC11976717; doi:10.1093/pnasnexus/pgaf087)
Supplement: pgaf087_Supplementary_Data [file pgaf087_supplementary_data.zip › PNASNEXUS-PNASNEXUS-2024-01121R-s05.pdf]

Electronic Supplemental Material for "Splash-free urinals for global sustainability and accessibility: design through physics and differential equations"

K. Thurairajah, X. Song, JD. Zhu,
Mia Shi, E. A. Barlow, R. C. Hurd, & Z. Pan

Table of Contents

1 Daily Urine Splash Estimation in the US **2**

2 Urination Apparatus and Experimental Setup **3**
2.1 Plumbing Overview 3
2.2 Nozzle Design 4
2.3 Critical Angle Testing Setup 5
2.4 Urinal Testing Setup 6

3 Test conditions **8**

4 High Speed Imaging and the Captions for the High Speed videos **9**
4.1 SI_video_1_disk_tests.mp4 9
4.2 SI_video_2_low_flow_urinal_tests.mp4 9
4.3 SI_video_3_med_flow_urinal_tests.mp4 9
4.4 SI_video_4_high_flow_urinal_tests.mp4 10

5 Theoretical Model for Splash Flow Rate Reduction through Angled Impact **10**

1 Daily Urine Splash Estimation in the US

We estimate the total volume of urine splashed onto floors, shoes, and pants in the United States. There are over $N = 56$ million urinals in non-residential settings in the US as of in 2019 [1]. The average male urinates V_u ~220 mL per urination and produces 6 voids during daytime, and 0.5 during the night [2]. Assuming that the non-residential nature of urinals leads to their use for 8 hours per day, and that the daytime lasts 16 hours and nighttime 8 hours, that leads to $v_d = 3$ to 6 voids per person per day. We also assume each urinal has on average $n_u = 1$ to 2 users. To estimate splash, we take advantage of the fact that most of the urinated volume is urinated at or near the peak flow rate [3]. Thus, we can use the test results in our research: $Q^* = 0.965\%$ for medium height high flow test value for the contemporary commercial urinal (see Fig. 6 in the main text). Multiplying these values together allows us to estimate the total splashed urine per day in the US:

$$\not{V} = N \cdot V_u \cdot v_d \cdot n_u \cdot Q^* \tag{S1}$$

$$= 56 \times 10^6\,\text{urinal} \;\cdot\; 0.22\,\frac{\text{L}}{\text{void}} \;\cdot\; (3,6)\frac{\text{void}}{\text{person day}} \;\cdot\; (1,2)\frac{\text{person}}{\text{urinal}} \;\cdot\; 0.965\% \tag{S2}$$

$$= (0.35, 1.4) \times 10^6\,\frac{\text{L}}{\text{day}} \tag{S3}$$

where $\not{V}$ gives the total splashed volume of urine in the US, and it is on the order of one million L/day (i.e., $\not{V} \sim O(10^6)$ L day^{-1}).

Given that the Nauti-loo design had only 1.4% of the splash of the contemporary commercial urinal (see Fig. 6 in the main text), we can estimate that the splash prevented as

$$\not{V}_p = \not{V} \cdot (1 - 1.4\%), \tag{S4}$$

where $\not{V}_p$ gives the total prevented urine splash volume in the US, and $\not{V}_p \sim O(10^6)$ L day^{-1} to be specific. If we assume that it takes ten times as much water to clean a certain volume of splashed urine, this implies that $O(10^7)$ L day^{-1} of fresh water would be saved for cleaning.

2 Urination Apparatus and Experimental Setup

2.1 Plumbing Overview

The urination apparatus is composed of a submersible pump within a large tank of water. The dimensions of the tank are 30 × 60 cm such that over the 1 L volume of the test the water level drops by only 0.55 cm. This water head change is negligible compared to the pressure provided by the pump and, thus the flow rate was able to be kept nearly constant. The pump is attached to a ball valve, which is used to start and stop the tests. Next, a flow rate meter is present with a totaliser to measure the flow, and a needle valve to adjust the flow rate. Finally, the nozzle generates a urine-like jet. The front end of the nozzle was treated with a hydrophobic coating to avoid the teapot effect and ensure that the flow cleanly detached from the nozzle.[1] Flexible tubing is used to connect the components.

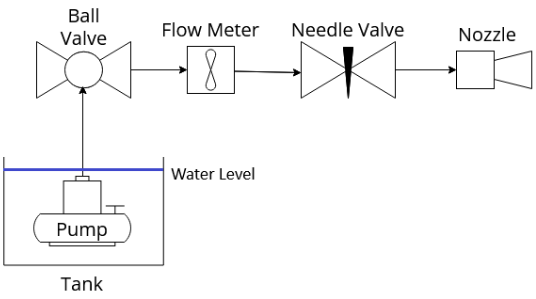

Fig. S1: Diagram illustrating the plumbing of the urination device.

[1]The hydrophobic coating is only needed at the low flow condition corresponding to a flow rate of 0.4 L/min

2.2 Nozzle Design

The nozzle of the urination device was 3D printed out of Acrylonitrile Butadiene Styrene (ABS) plastic, and threads were cut with a 1/2 inch National Pipe Taper (NPT) tap. The inner geometry starts out with a 1/2 inch NPT circular cross-section with a smooth transition to an elliptical cross-section. The elliptical orifice is based on the geometry of a typical human urethra, which is important to the dynamics of the urine jet (i.e., the capillary waves and the breaking up of the jet into the droplet train) [4]. The front end of the nozzle was treated with a hydrophobic coating to ensure that the flow cleanly detached from the nozzle even at low flow rates.

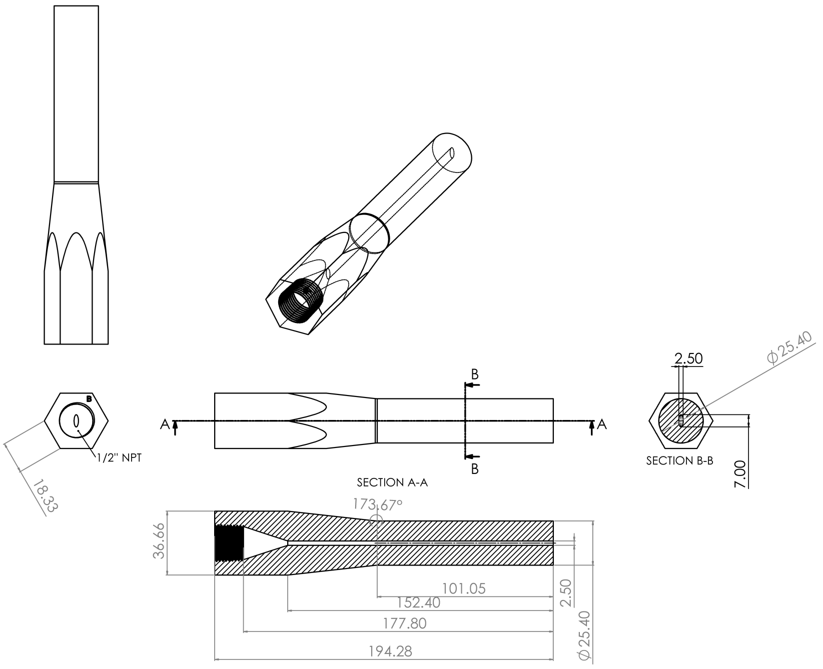

Fig. S2: Technical drawing of the anatomically accurate nozzle of the urination apparatus.

2.3 Critical Angle Testing Setup

The nozzle was set up vertically for the critical angle tests with the long axis of the orifice oriented horizontally when projected onto the view shown in Fig. S3. The vertical stream impinges on the angled glass plate within the bucket as is seen in the figure below. The angle was measured using a Digital Angle Indicator. The 10 cm diameter disk was placed within a bucket with an inner diameter of 18.5 cm. Flow rates were adjusted prior to testing. The paper towel was mounted on supporting structures placed around the bucket to absorb any splash. The mass of the paper towel was measured before and after testing using an analytical scale (AAA 250L, Adam Equipment, USA). The vertical height between the nozzle and the glass disk (h) is measured from the nozzle outlet to the center of the disk.

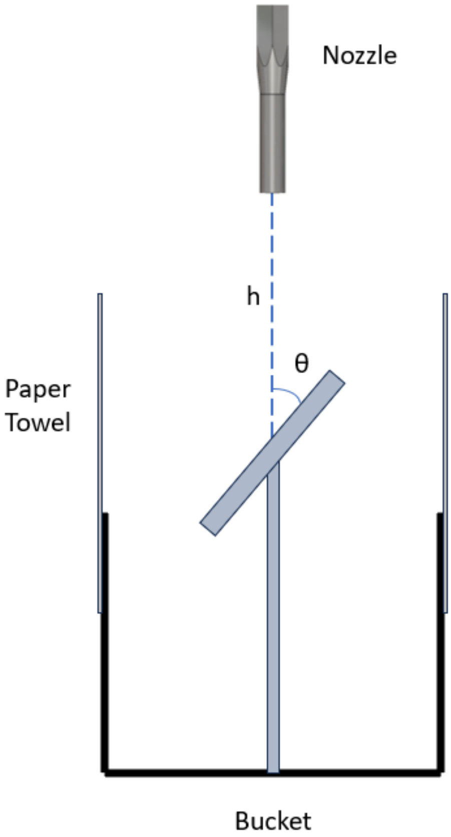

Fig. S3: A diagram of the critical angle testing. h indicates the nozzle height above the glass disk. θ indicates the impinging angle.

2.4 Urinal Testing Setup

The urination apparatus was set up with a horizontally oriented nozzle to test the urinals. The nozzle was centered widthwise, and the height was based on anthropometric data and was adjusted using extrusions [5]. The tip was placed 2 cm in front of the lip of the urinal to ensure that all drops would fall within the urinal, which also reflects a proper manner of using a urinal. The orifice was oriented with the long axis oriented vertically. The urinal was mounted on a frame constructed of aluminum extrusions. The height of the urinal installation was based on either its design for the novel urinals, or installation standards for the existing ones [6]. A frame of steel wire was placed on the outside of the urinal to mount paper towels enclosing the inner surface which catch droplets splashed from the urinal.

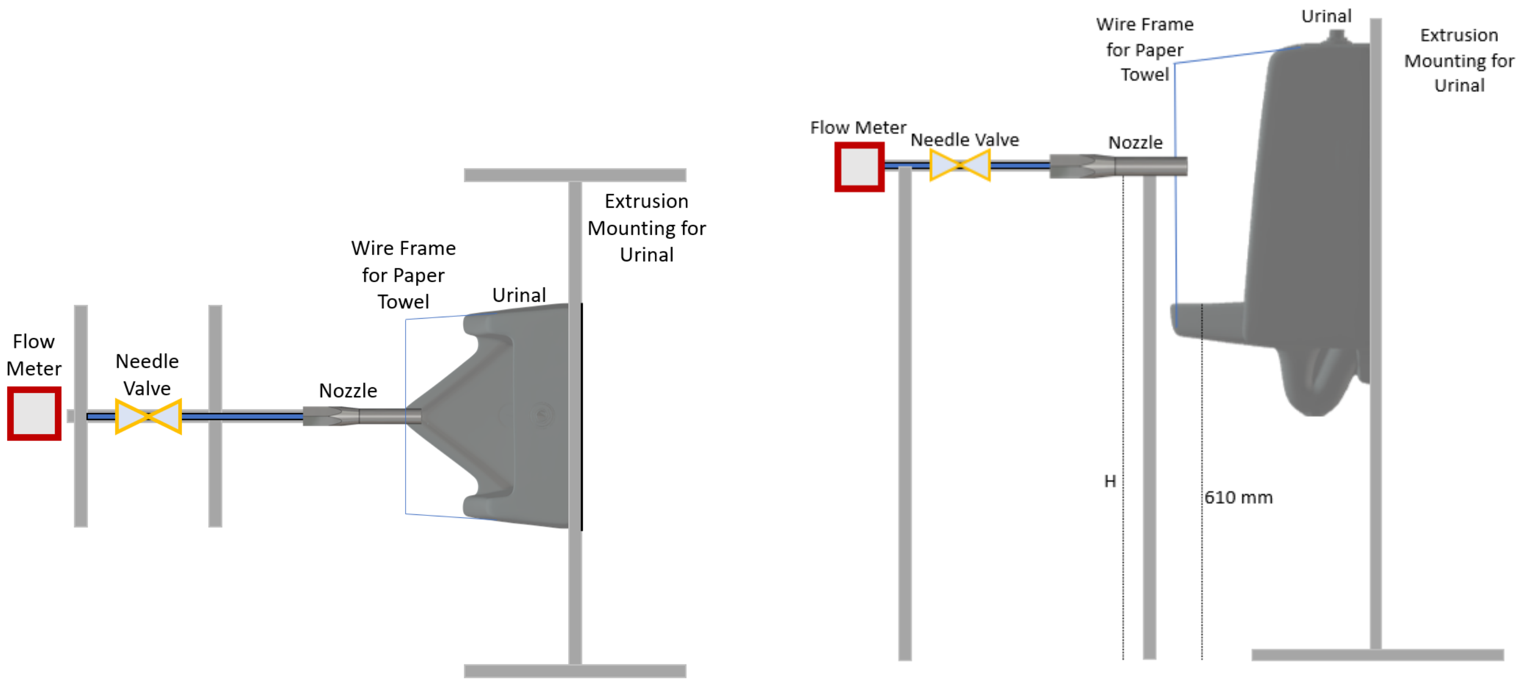

Fig. S4: A diagram showing the setup for experiments measuring splash from urinals (not to scale). The urinal is mounted on a frame with wires to support the paper towel which catches the splash. H indicates the nozzle height above the ground (left image: top view, right image: side view).

3 Test conditions

Flow rates in the tests encompassed what could be reasonably expected from the typical male human population, with a $Q_{max} = 1.9$ L/min, representing the 95th percentile peak flow rate for men under 50 with an average voided volume; $Q_{median} = 0.7$ L/min representing median average flow rate in a similar case, and a $Q_{min} = 0.4$ L/min representing a bound well below the 5th percentile average flow rate and associated with the lowest value measured by the flow rate meter used [7]. The high flow rate represented a condition which although high was still reasonable to be encountered by a public urinal. The low flow rate was set as low as possible rather than a fixed percentile. As urination progresses flow rate drops steadily, thus although a high flow rate is rarely encountered, a low flow rate is always encountered. The low flow rate explores one extreme and impinges on different regions of the urinals.

In conducting critical impinging angle testing (Section 5.3 in the main text), the jet travelled a vertical distance prior to impacting the glass plate, with the low height being equivalent to the distance from 5th percentile crotch height to the lip of a standard urinal, the medium being equivalent to the distance from 50th percentile crotch height to lip height, and the high height condition being from 95th percentile crotch height to the ground [5, 6].

A series of images was taken during tests and used to determine the distribution of droplet sizes in the stream immediately prior to impact. The median droplet sizes, associated We and the test conditions are listed in Table 1.

Table 1: Test conditions for critical impinging angle testing. The indicated height is the vertical distance between the nozzle outlet and the center of the glass disk. The indicated We is associated with the median droplet diameter.

Test Condition	Nozzle Height: h (mm)	Flow Rate (L/min)	Median Droplet Diameter (mm)	We
High Height Medium Flow	933	0.7	5.5	1400
Medium Height Low Flow	254	0.4	4.4	340
Medium Height Medium Flow	254	0.7	5.9	450
Medium Height High Flow	254	1.9	7.2	580
Low Height Medium Flow	184	0.7	5.4	370

For the urinal testing (Section 5.5 in the main text), all tests were conducted with a nominal water volume of 1 L. The low, medium, and high heights are based on 5th, 50th, and 95th percentile inseam heights respectively for American males. The test conditions are listed in Table 2.

Table 2: Test Matrix for urinal testing. The indicated height is the height of the nozzle above the ground.

Test Condition Name	Nozzle Height: H (mm)	Flow Rate (L/min)
High Height Medium Flow	933	0.7
Medium Height Low Flow	864	0.4
Medium Height Medium Flow	864	0.7
Medium Height High Flow	864	1.9
Low Height Medium Flow	794	0.7

4 High Speed Imaging and the Captions for the High Speed videos

High-speed video footage was taken of the urinals under the same experimental conditions as was used for the splatter experiment. The high-speed camera (Chronos 2.1 HD, Kron Technologies, Canada) recorded at 1,512 frames per second (FPS) with a resolution of 1280 × 1024 pixels.

4.1 SI_video_1_disk_tests.mp4

A high-speed video depicting the tests used to measure the critical angle as described in (Section 5.3 in the main text). In this case, the absorbent towel is removed to allow the splash to be imaged by a high-speed camera at 1,512 FPS and replayed at 60 FPS. Three impinging angles are shown (left to right: 90°, 60°, and 30°, respectively).

4.2 SI_video_2_low_flow_urinal_tests.mp4

A compilation of high-speed videos depicting the urinal splash tests as described in (Section 5.5 in the main text). In this case, the absorbent towel is removed to allow the splash to be imaged by a high speed camera at 1,512 FPS and replayed at 60 FPS. All tests are conducted with the nozzle at median inseam height, with low flow rate. Existing designs follow the ASME standard urinal installation and intended installation for the novel ones. Different urinals are displayed, from left to right: a) contemporary commercial, b) La Fontaine, c) Nauti-loo, and d) Cornucopeea.

4.3 SI_video_3_med_flow_urinal_tests.mp4

A compilation of high-speed videos depicting the urinal splash tests as described in (Section 5.5 in the main text). In this case, the absorbent towel is removed to allow the splash to be imaged by a high-speed camera at 1,512 FPS and replayed at 60 FPS. All tests are conducted with the nozzle at median inseam height, with medium flow rate. Existing designs follow ASME standard urinal installation and intended installation for the novel ones. Different urinals are displayed, from left to right: a) contemporary commercial, b) La Fontaine, c) Nauti-loo, and d) Cornucopeea.

4.4 SI_video_4_high_flow_urinal_tests.mp4

A compilation of high-speed videos depicting the urinal splash tests as described in (Section 5.5 in the main text). In this case, the absorbent towel is removed to allow the splash to be imaged by a high-speed camera at 1,512 FPS and replayed at 60 FPS. All tests are conducted with the nozzle at median inseam height, with high flow rate. Existing designs have ASME standard urinal installation and intended installation for the novel ones. Different urinals are displayed, from left to right: a) contemporary commercial, b) La Fontaine, c) Nauti-loo, and d) Cornucopeea.

5 Theoretical Model for Splash Flow Rate Reduction through Angled Impact

Hao et. al (2019) showed experimentally, and modeled theoretically, the phenomenon of splash suppression for droplets impinging on angled surfaces [8]. They determined that splash occurs if the lamellar velocity is high enough to generate lift, specifically if it is higher than a threshold. This threshold lamellar velocity was defined as the lamellar velocity for an orthogonal impact at a threshold impact velocity. The threshold impact velocity for orthogonal impact is the minimum velocity required to generate splash when a droplet impacts a surface. However, this work and many other relevant works [9, 10, 11] often focus on developing the criteria predicting splash onset instead of estimating the flow rate of splash, which is the key for the purpose of the current research. Thus, we built upon the principles of the model in [8] and extended their work to predict the change in splash fraction rate when varying the impinging angle.

Similar to Hao et. al (2019), we assume a certain threshold must be met, below which no splash occurs. We also assume that the mass of the lifted lamella, indicating the splash flow rate, is proportional to the aerodynamic lift surplus to this threshold. This is a simple assumption that more lift is required to splash more mass. The change in lamellar velocity with respect to the threshold reflects how the splash flow rate is affected by impact angle and speed. The aerodynamic lift was quantified by averaging the difference between the square of the lamella velocity and the square of the threshold lamella velocity over the radial angle. This averaging is necessary as the lamella velocity is higher in some areas and lower in others.

In the work by Hao et. al (2019) the expression for the lamella velocity V_{ln} for a normal impact is given by:

$$V_{ln} = \frac{\sqrt{3}}{2}\sqrt{D_0 V_n / 2T}, \tag{S5}$$

where D_0 is the droplet diameter, V_n is the normal impact velocity, and T the time [8]. The velocity is highest at the moment of lamella formation T_e and is given by:

$$T_e = \frac{D_0(1.1\sqrt{We})^{-4/3}}{2V_n}, \tag{S6}$$

where $We = \frac{\rho V_0^2 D_0}{\gamma}$ is the Weber number, V_0 is the droplet velocity, D_0 is the droplet diameter, ρ is the density of the liquid, and γ is the surface tension of

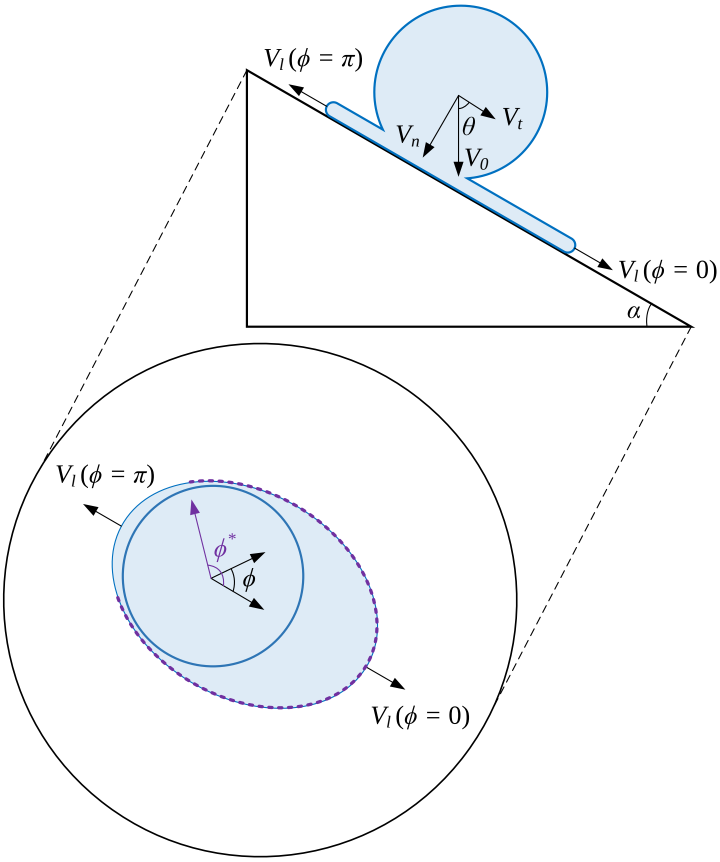

Fig. S5: A digram showing the impact of a droplet on an angled surface and the resultant lamella moving outwards. The upper image is a side view and the lower image is a top view, orthogonal to the impinging plane. The radial angle ϕ is also shown. Due to V_t, V_l varies with ϕ, at $\phi = 0$ the tangential velocity of the droplet adds to the lamellar velocity and at $\phi = \pi$ it is subtracted. This means that for certain angles V_l is too low to generate splash. The dotted purple line shows the range of radial angles where splash can be generated. If $\phi > \phi^*$ no splash occurs.

the liquid. The lamellar velocity of the angled droplet impact is given as

$$V_l(\alpha, \phi) = V_{ln}(\alpha) + V_t(\alpha)\cos\phi, \tag{S7}$$

where $V_n = V_0 \cos\alpha$ and $V_t = V_0 \sin\alpha$ are the normal and tangential impact speeds, respectively (shown in Fig. S5). The second term in (S7) gives the tangential component of the droplet velocity added to the lamella [8].

At the time of lamella formation, letting $T = T_e$, substituting (S5) and (S6)

in (S7) leads to:

$$
\begin{aligned}
V_l(\alpha, \phi) &= \frac{\sqrt{3}}{2}\sqrt{D_0 V_0 \cos\alpha / 2T_e} + V_0 \sin\alpha\cos\phi \\
&= \frac{\sqrt{3}}{2} V_0 \cos\alpha (1.1\sqrt{We})^{2/3} + V_0 \sin\alpha \cos\phi.
\end{aligned}
\tag{S8}
$$

It should be noted that $\alpha = \pi/2 - \theta$, where θ is the impinging angle.

The scaling of the averaged surplus lift is obtained by integrating the surplus lamellar velocity over the radial angle ϕ. The integral takes advantage of the symmetry of the problem and is performed from $\phi = 0$ to $\phi = \phi^* \leq \pi$. If the velocity is below the threshold it contributes no splash, and does not count towards the average. ϕ^* is the angle where the lamellar velocity is equal to the threshold as seen in Fig. S5. Beyond this angle the velocity is not sufficient to generate lift and as such this portion of the lamella does not contribute to the splash. ϕ^* can be computed by setting

$$V_l^* = V_l(\phi^*),$$

where $V_l^* = \frac{\sqrt{3}}{2} V^* \beta^{*2/3}$ and $V_l(\phi^*) = \frac{\sqrt{3}}{2} V_0 \cos\alpha \beta^{2/3} + V_0 \sin 2\alpha \cos\phi^*$, respectively:

$$\frac{\sqrt{3}}{2} V^* v^{*2/3} \beta^{2/3} = \frac{\sqrt{3}}{2} V_0 \cos\alpha\beta^{2/3} + V_0 \sin\alpha\cos\phi^*. \tag{S9}$$

Solving ϕ^* from (S9), we have

$$\phi^* = \Re\left[\cos^{-1}\left(\sqrt{3}\beta^{2/3}\frac{v^{*5/3} - \cos\alpha}{2\sin\alpha}\right)\right] = \Re\left[\cos^{-1}(\xi)\right], \tag{S10}$$

where $\xi = \sqrt{3}\beta^{2/3}\frac{v^{*5/3} - \cos\alpha}{2\sin\alpha}$. Taking the real part of $\cos^{-1}(\xi)$ in (S13) guarantees that $\phi^* \in [0, \pi]$, no matter how ξ varies. This recovers the physical intuition beyond the mathematical practice. For example, for high impinging angles (high θ and small α) the lamellar velocity is always above the threshold for all ϕ. In this case, ϕ^* saturates at π, and we should be integrating across the entire semicircle.

The scaling of the average surplus lift therefore is given by the following integral:

$$Q^* \sim \langle L_s \rangle \sim \frac{1}{\pi}\int_0^{\pi} \max\left(V_l^2 - V_l^{*2} 0\right) d\phi = \frac{1}{\pi}\int_0^{\phi^*} V_l^2 - V_l^{*2}\, d\phi, \tag{S11}$$

where Q^* is the non-dimensional splash ratio given by $Q^* = m'/m$, where m' is splashed mass and m is total impinged mass. $\langle L_s \rangle$ is the angular average surplus lift. V_l^* is a threshold lamellar velocity given by substituting the threshold normal impact velocity, and $\alpha = 0$ into (S8):

$$V_l^* = \frac{\sqrt{3}}{2} V^* (1.1\sqrt{We^*})^{2/3}, \tag{S12}$$

where V^* is the critical splash velocity for a droplet impinging normally to a surface ($\alpha = 0$). This is to say that if a droplet is travelling at $V_0 < V^*$ and

impacts normal to a surface splash will not occur, and if $V_0 > V^*$ splash will occur. We^* is the critical Weber number associated with V^*. For a concise presentation of the equations, we denote $\beta = 1.1\sqrt{We}$ and $\beta^* = 1.1\sqrt{We^*} = v^*\beta$, where we set $v^* = V^*/V_0$ as the non-dimensional critical velocity. Substituting (S8), (S10), and (S12) into the integral given in (S11) obtains:

$$\begin{aligned} Q^* &\sim \int_0^{\phi^*} V_0^2 \left(\frac{\sqrt{3}}{2}\cos\alpha\beta^{2/3} + \sin\alpha\cos\phi\right)^2 - V^{*2}\left(\frac{\sqrt{3}}{2}\beta^{*2/3}\right)^2 d\phi \\ &= \frac{V_0^2}{\pi}\left[\frac{3}{4}\cos^2\alpha\beta^{4/3}\phi^* + \frac{1}{2}\sin^2\alpha(\phi^* + \frac{1}{2}\sin 2\phi^*)\right. \\ &\quad \left. + \frac{\sqrt{3}}{2}\sin 2\alpha\beta^{2/3}\sin\phi^* - \frac{3}{4}v^{*2}\beta^{*4/3}\phi^*\right]. \end{aligned} \tag{S13}$$

The estimate of Q^* in (S13) can be normalized by the flow rate of perpendicular impact. This practice allows us to study the splash fraction changes with impinging angle, which serves the purpose of the current research. The normalisation factor is calculated by setting $\alpha = 0$, which is equivlent to $\theta = 90°$ impinging angle, and $\phi^* = \pi$ in (S13) giving:

$$Q^*_{90°} \sim \frac{3}{4}\left(V_0^2\beta^{4/3} - V^{*2}\beta^{*4/3}\right). \tag{S14}$$

Normalizing the flow rate Q^* by dividing (S13) by (S14), and invoking $\theta = \pi/2 - \alpha$, the normalized splash fraction is given below:

$$\frac{Q^*}{Q^*_{90°}} = \frac{1}{\pi} + \frac{\phi^*\left(\cos^2\theta\left(2 + \sin 2\phi^* - 3\beta^{4/3}\right) + \frac{2\sqrt{3}}{\phi^*}\sin 2\theta\beta^{2/3}\sin\phi^*\right)}{3\pi\beta^{4/3}\left(1 - v^{*\frac{10}{3}}\right)}, \tag{S15}$$

where

$$\phi^* = \Re\left[\cos^{-1}\left(\sqrt{3}\beta^{2/3}\frac{v^{*5/3} - \sin\theta}{2\cos\theta}\right)\right].$$

This model however has a critical inconsistency. The high-speed thin lamella is mainly formed from the normal component of the droplet velocity V_n yet the expression for lamellar velocity depends on the Weber number associated with V_0. Unlike the reference [8], which used We based on V_0, we propose to use a modified Weber number $We^\dagger$ based on the normal portion of velocity V_n as follows:

$$We^\dagger = \frac{\rho V_n^2 l}{\gamma} = We\cos^2\alpha \tag{S16}$$

$$\beta^\dagger = 1.1\sqrt{We^\dagger} = \beta\cos\alpha. \tag{S17}$$

For a normal impact $We^\dagger = We$ and thus (S14) is identical regardless of if the modified Weber number is used. Similar to the derivation for (S9), we obtain an alternative expression for ϕ^*. In this case, the left hand side remains the same as the threshold condition is for a normal impact and We are equivalent with

or without modification. On the right hand side, the lamellar velocity is now a function of the modified Weber number $We^\dagger$ and as such uses $\beta^\dagger$:

$$\frac{\sqrt{3}}{2}V^*\beta^{*2/3} = \frac{\sqrt{3}}{2}V_0\cos\alpha{\beta^\dagger}^{2/3} + V_0\sin\alpha\cos\phi^* \tag{S18}$$

and

$$\phi^* = \Re\left[\cos^{-1}\left(\sqrt{3}\beta^{2/3}\frac{v^{*5/3}-\cos^{5/3}\alpha}{2\sin\alpha}\right)\right]. \tag{S19}$$

If the modified Weber number $We^\dagger$ is used, the normalised splash fraction is given by the following expression:

$$\begin{aligned}\frac{Q^*}{Q^*_{90^\circ}} &= \frac{1}{\pi}\frac{\sin\theta - v^{*10/3}}{1-v^{*10/3}}\\ &+ \frac{\phi^*\left(\cos^2\theta\left(2+\sin 2\phi^* - 3{\beta^\dagger}^{4/3}\right) + \frac{2\sqrt{3}}{3\phi^*}\sin 2\theta{\beta^\dagger}^{2/3}\sin\phi^*\right)}{3\pi\beta^{4/3}\left(1-v^{*10/3}\right)},\end{aligned} \tag{S20}$$

where

$$\phi^* = \Re\left[\cos^{-1}\left(\sqrt{3}\beta^{2/3}\frac{v^{*5/3}-\sin^{5/3}\theta}{2\cos\theta}\right)\right].$$

The use of a modified Weber number aligns with the physical intuition of the problem: When a droplet impacts a surface the normal component contributes to the generation of high-speed lamella, with the tangential velocity component simply adding an overall velocity. This simple modification to the model corresponds to much better agreement with experiments, indicating a better representation of the underlying phenomena (see Fig. S7).

The threshold velocity V^* represents the criterion between splash and no-splash for a perpendicular impact. Vander Wal et. al. (2005) showed that this critical value is $\sqrt{Ca} = 0.35$; where $Ca = \frac{\mu V}{\gamma}$ is the capillary number [12]. Importantly, the dynamic viscosity μ and surface tension γ do not change across our experiments, nor in the context of human urination. Thus, a critical velocity V^* fully determines a critical Ca^*.

Adopting the critical $Ca^* = 0.35^2$ from reference [12], corresponding to $V^* = 0.89$ m/s, we plot our theoretical model (S20) against experimental data (see Section 2.3 in the Supplementary Material). The centerlines of the patches in Fig. S6 are evaluated by equation (S20) based on the median droplet diameter. The upper and lower edges of the patches indicate the varied $We^\dagger$ based on plus or minus one standard deviation of droplet size. The median droplet diameter is used to normalize the upper and lower boundaries as shown in equation. (S14). The statistics of the droplet sizes were measured from images of the experimental tests, while velocities were calculated from the nozzle outlet area, flow rate, and acceleration due to gravity. The model using the modified We fits the experimental data well for every flow condition (see Fig. S6).

Fig. S7(a) summarises the results shown in Fig. S6, highlighting the invariance of the critical angle across test cases. The centerline is omitted for clarity. The model based on the unmodified We, (S15), is similarily shown Fig. S7(b). The theoretical prediction based on unmodified We significantly overestimates

for low impact angles. However, the model based on $We^\dagger$ provides a significant improvement to the fit. Notably, no threshold for the unmodified We provides as good a fit as the modified $We^\dagger$ model. If the threshold is increased to account for the overestimation of splash the invariance of the critical angle is lost.

(a)

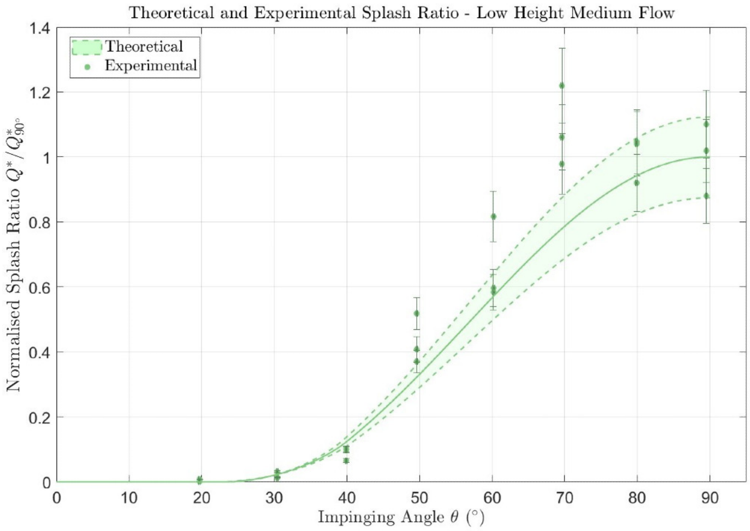

(b)

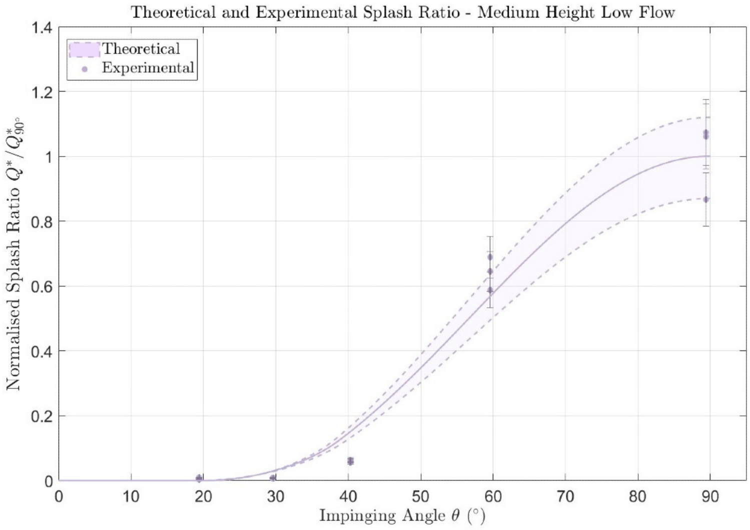

(c)

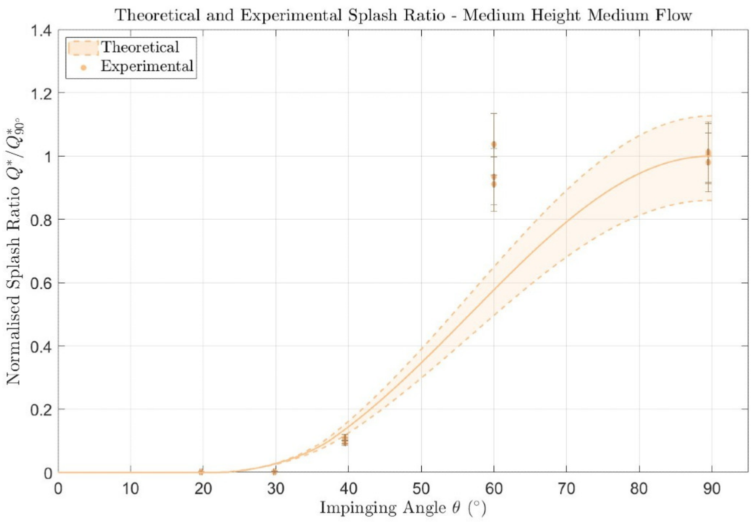
Theoretical and Experimental Splash Ratio - Medium Height Medium Flow
Theoretical
Experimental
Normalised Splash Ratio $Q^*/Q^*_{90°}$
Impinging Angle θ (°)
0
0.2
0.4
0.6
0.8
1
1.2
1.4
0
10
20
30
40
50
60
70
80
90

(d)

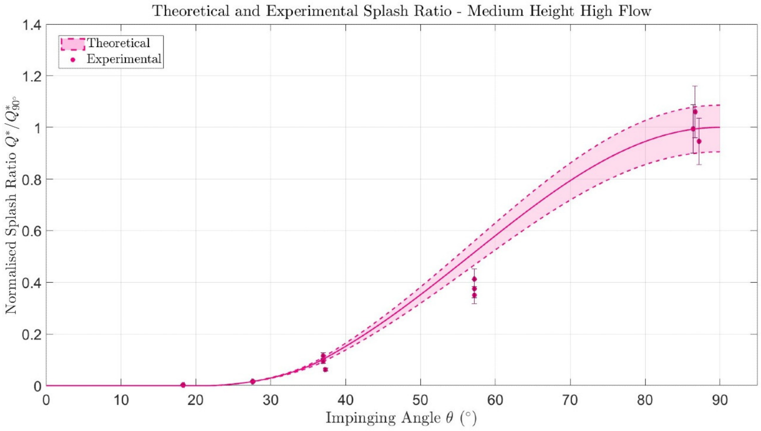
Theoretical and Experimental Splash Ratio - Medium Height High Flow
Theoretical
Experimental
Normalised Splash Ratio $Q^*/Q^*_{90°}$
Impinging Angle θ (°)
0
0.2
0.4
0.6
0.8
1
1.2
1.4
0
10
20
30
40
50
60
70
80
90

(e)

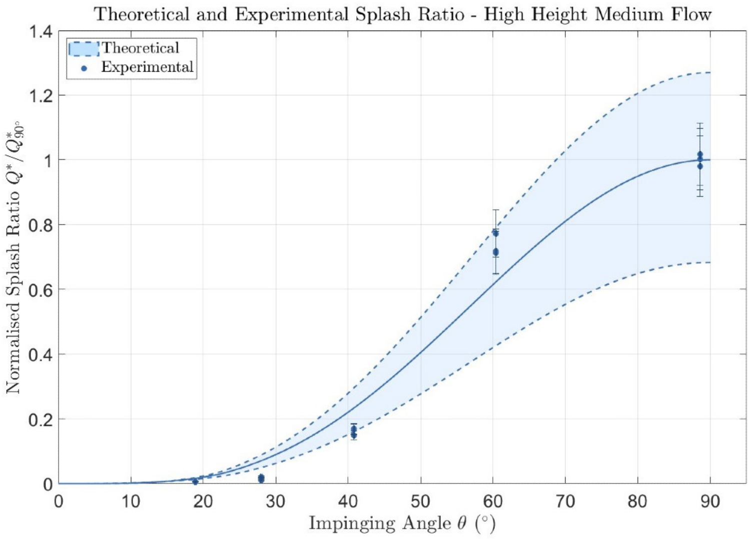

Fig. S6: Plots individually comparing theoretical and experimental results for cases spanning the expected conditions for human urination. All models are based on the modified Weber number $We^\dagger$. (a) shows results for Low Height Medium Flow, (b) shows Medium Height Low Flow, (c) shows Medium Height Medium Flow (d) Medium Height High Flow. and (e) shows High Height Medium Flow. Flow conditions are specifically outlined in section. 2.3.

(a)

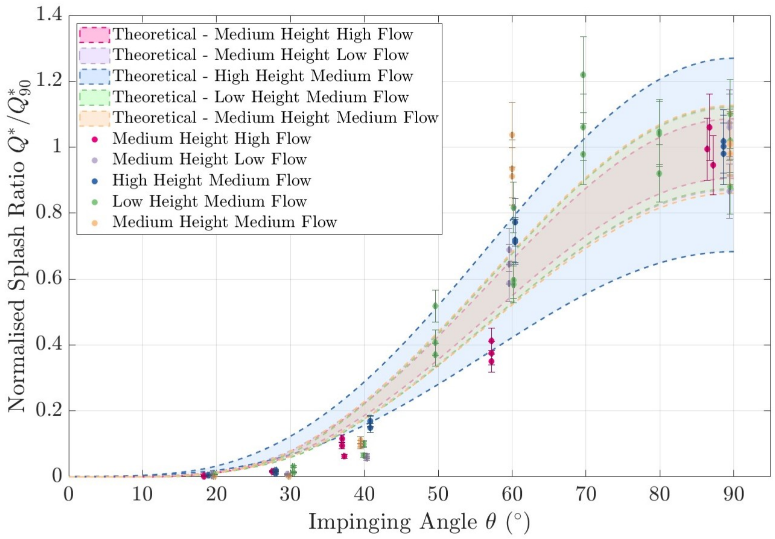

(b)

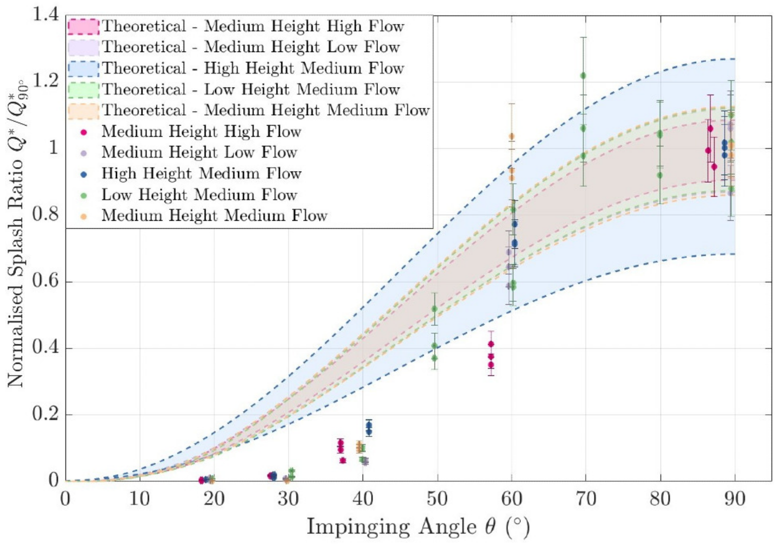

Fig. S7: Plots comparing theoretical and experimental normalized splash ratio with the $\sqrt{Ca} = 0.35$ threshold for both the unmodified and modified Weber numbers. (a) shows the modified $We^{\dagger}$ model and (b) shows the model without the modified We number.

References

[1] GMP Research Inc. 2019 u.s. watersense market penetration. https://www.safeplumbing.org/files/safeplumbing.org/documents/misc/7-1-19-WaterSense-2019-Report.pdf, Jun 2019.

[2] B. Folkestad and A. Spångberg. Timed micturition and maximum urinary flow rate in randomly selected symptom-free males. *Scandinavian Journal of Urology and Nephrology*, 38:136–142, 2004.

[3] J. Chung and R. v. Mastrigt. Age and volume dependent normal frequency volume charts for healthy males. *Journal of Urology*, 182:210–214, 2009.

[4] Andrew PS Wheeler, Samir Morad, Noor Buchholz, and Martin M Knight. The shape of the urine stream-from biophysics to diagnostics. 2012.

[5] *Anthropometry and Biomechanics.* NASA, July 2018. Available: https://msis.jsc.nasa.gov/sections/section03.htm [Last accessed: 17 September 2023].

[6] America Inc. America Standard. Installation instructions washbrook urinal 6590 & 6515 series, 2018. Online; accessed 29 January 2023.

[7] BT Haylen, Deborah Ashby, JR Sutherst, MI Frazer, and CR West. Maximum and average urine flow rates in normal male and female populations-the liverposl nomograms. *British journal of urology*, 64(1):30–38, 1989.

[8] Jiguang Hao, Jie Lu, Liaonan Lee, Zhihu Wu, Gengkai Hu, and JM Floryan. Droplet splashing on an inclined surface. *Physical review letters*, 122(5):054501, 2019.

[9] A. L. Yarin. Drop impact dynamics: splashing, spreading, receding, bouncing.... *Annual Review of Fluid Mechanics*, 38:159–192, 2006.

[10] G. Cossali, A. Coghe, and M. Marengo. The impact of a single drop on a wetted solid surface. *Experiments in Fluids*, 22:463–472, 1997.

[11] R. L. V. Wal, G. M. Berger, and S. D. Mozes. The splash/non-splash boundary upon a dry surface and thin fluid film. *Experiments in Fluids*, 40:53–59, 2005.

[12] R. L. V. Wal, G. M. Berger, and S. D. Mozes. The splash/non-splash boundary upon a dry surface and thin fluid film. *Experiments in Fluids*, 40:53–59, 2005.
